# Hierarchical Micro-Nano Topography Promotes Cell Adhesion and Osteogenic Differentiation via Integrin α2-PI3K-AKT Signaling Axis

**DOI:** 10.3389/fbioe.2020.00463

**Published:** 2020-05-19

**Authors:** Huimin Zheng, Yujuan Tian, Qian Gao, Yingjie Yu, Xianyou Xia, Zhipeng Feng, Feng Dong, Xudong Wu, Lei Sui

**Affiliations:** ^1^Department of Prosthodontics, School and Hospital of Stomatology, Tianjin Medical University, Tianjin, China; ^2^Department of Cell Biology, 2011 Collaborative Innovation Center of Tianjin for Medical Epigenetics, Tianjin Key Laboratory of Medical Epigenetics, Tianjin Medical University, Tianjin, China; ^3^Health Science Center, Institute of Translational Medicine, The First Affiliated Hospital of Shenzhen University, Shenzhen, China

**Keywords:** topography, adhesion, osteogenic differentiation, integrin α2, PI3K-AKT

## Abstract

Surface topography dictates important aspects of cell biological behaviors. In our study, hierarchical micro-nano topography (SLM-AHT) with micro-scale grooves and nano-scale pores was fabricated and compared with smooth topography (S) and irregular micro-scale topography (SLA) surfaces to investigate mechanism involved in cell-surface interactions. Integrin α2 had a higher expression level on SLM-AHT surface compared with S and SLA surfaces, and the expression levels of osteogenic markers icluding Runx2, Col1a1, and Ocn were concomitantly upregulated on SLM-AHT surface. Moreover, formation of mature focal adhesions were significantly enhanced in SLM-AHT group. Noticablely, silencing integrin α2 could wipe out the difference of osteogenic gene expression among surfaces with different topography, indicating a crucial role of integrin α2 in topography induced osteogenic differentiation. In addition, PI3K-AKT signaling was proved to be regulated by integrin α2 and consequently participate in this process. Taken together, our findings illustrated that integrin α2-PI3K-AKT signaling axis plays a key role in hierarchical micro-nano topography promoting cell adhesion and osteogenic differentiation.

## Introduction

Surface topography is a key determinant of the cellular response to foreign materials (Chen et al., 2014; Dalby et al., 2014; Gautrot et al., 2014; Denchai et al., 2018), which is extremely important for intraosseous implants to achieve osseointegration. In the past few years, there has been growing interest in the effects of different surface features at various scales on cell adhesion, proliferation, and osteogenic differentiation (Anselme et al., 2010; Chen et al., 2014; Li et al., 2016; Skoog et al., 2018; Zhang et al., 2019). It is well recognized that micro-scale structures ensure the initial stability of the implant and promote the bone locking (Saruta et al., 2019), while nano-scale structures have more significant effects on the adhesion and differentiation of cells (Kim et al., 2013; Gorelik and Gautreau, 2014; Cimmino et al., 2018). Furthermore, it has been illustrated that the regulatory effect of nano-scale structures is precisely based on the mechanical retention provided by the micro-scale structures, i.e., the initial stability of intraosseous implants (Deng et al., 2019). Accordingly, hierarchical micro-nano topography is a better choice for intraosseous implants in mediating cell-surface interactions compared to single-scale topography. It is noteworthy that natural bone is a loose porous multi-ordered structure composed of nano-scale collagen and hydroxyapatite and micro-scale bone plates and pores (Robling et al., 2006; Karsenty et al., 2009; Zhu et al., 2020). To mimic this environment, it is also of great significance to manufacture intraosseous implants with hierarchical micro-nano surface topography (Shah et al., 2018; Cui et al., 2019; Zhang et al., 2019). In our previous work, we fabricated titanium surfaces with hierarchical microprotrusion-nanonotch topography using direct metal laser sintering technique together with acid etching treatment, which could promote osteogenic differentiation of stem cells (Zheng et al., 2018). To enable further performance improvements, hierarchical microgroove-nanopore topography was fabricated by selective laser melting (SLM) technique combined with alkali heat treatment (AHT) because of higher fabricating efficiency and less consumption of raw materials. And our preliminary results indicated that this topography could better promote cell adhesion and osteogenic differentiation. However, the mechanisms implicated in the cell-surface interactions remain unclear.

In the early stages of the cell-surface interactions, cells sense the environment through lamellipodia and filopodia composed of integrins (Mattila and Lappalainen, 2008; You et al., 2014; Sun et al., 2016; Michael and Parsons, 2020). During spreading on the surface, cells initially form nascent adhesions (NAs) at their periphery (Bachir et al., 2014). Some of NAs mature into focal complexes (FCs) (Sun et al., 2014). Both NAs and FCs are not stable. They either disappear in minutes or transform into focal adhesions (FAs) (Valdembri and Serini, 2012), which consist of several proteins including integrin, vinculin, talin, paxillin, tensin, zyxin, focal adhesion kinase (FAK), and α-actinin (Wozniak et al., 2004), and anchor cells to the substrate (Li et al., 2017). It was reported that by simply controlling the topography of surfaces, one can modulate the formation of FAs, and consequently alter cell-surface interactions (Geiger et al., 2009; Nasrollahi et al., 2016; Lou et al., 2019; Janssen et al., 2020). Integrin, one of the indispensable component of FAs, is likely to be involved in this process (Ginsberg, 2014; De Franceschi et al., 2015; Karimi et al., 2018). Various integrins consisting of different α and β subunits are recruited on different surface topography (De Franceschi et al., 2015). Among them, integrin α2 is considered playing an important role in regulating cell adhesion and osteogenic differentiation (Olivares-Navarrete et al., 2015). Knockdown of α2 integrin subunits inhibited the formation of osteogenic microenvironment (Raines et al., 2019). And it was also reported that integrin α2 is required for activation of Runx2, the following expression of Ocn, and ECM mineralization procedure (Siebers et al., 2005; Hui-Min, Hu et al., 2013). Accordingly, we hypothesize that integrin α2 expression could be regulated by surface structures and subsequently influence osteoblast differentiation.

The osteogenic promoting effect of integrin α2 is possibly related to PI3K-AKT signaling which is involved in multiple cell behaviors including proliferation, apoptosis, glucose metabolism, differentiation and migration (Gu et al., 2013; Edlind and Hsieh, 2014; Feng et al., 2018). PI3Ks are composed of a regulatory subunit (p85) and a catalytic subunit (p110), and can be activated via tyrosine kinase and cytosolic tyrosine kinases (Xu et al., 2015; Arienti et al., 2019). Recent study showed that integrin α2 could active PI3K by enhancing phosphorylation of FAK (p397 FAK) (Yoon et al., 2017), and upregulation of PIP2 would result in loss of FAs (Izard and Brown, 2016). In addition, PI3K-AKT signaling and Runx2 were demonstrated mutually dependent on each other in the regulation of cell differentiation (Chuang et al., 2013; Choi et al., 2014). Taken all aspects above, we suppose that hierarchical micro-nano topography upregulates integrin α2 expression, which in turn activates PI3K-AKT signaling and thereby promotes osteogenic differentiation.

This study aims to investigate the role of integrin α2-PI3K-AKT signaling axis in hierarchical micro-nano topography induced cell adhesion and osteogenic differentiation. SLM-AHT titanium surfaces were fabricated and characterized, machined (S) and conventional sand-blasted, large grit and acid etching (SLA) titanium surfaces were used as control. To understand the effect of micro-nano topography on FAs, we stained the cells for vinculin, performed image analysis, and measured the numbers and size of the FAs on three surfaces. Meanwhile, in order to elucidate the role of integrin α2, we knockdown and overexpress integrin α2 in MC3T3-E1 cell line. Our results indicate that hierarchical micro-nano topography could promote cell adhesion by enhancing assembling of mature FAs through increased expression of integrin α2. Furthermore, PI3K-AKT signaling is also influenced by the expression of integrin α2 while consequently regulates osteogenic differentiation, therefore, integrin α2-PI3K-AKT signaling axis plays a key role in hierarchical micro-nano topography promoting cell adhesion and osteogenic differentiation.

## Materials and Methods

### Specimen Preparation

Three groups of titanium specimens (disk-shaped, 6-mm in diameter and 2-mm in thickness, Ti-6Al-4V; Institute of Aeronautical Materials, Beijing, China) were prepared, including SLM-AHT group, SLA group and smooth titanium (S) group. SLM-AHT disks were fabricated in an argon atmosphere with Yb fiber laser system (EOS M280, EOS GmbH, Krailling, Germany) using a wave length of 1054 nm, continuous power of 200 W, scanning speed of 7 m-s and a laser spot size of 0.1 mm. The resultant disks were then etched with 1.5% HF for 30 min. After that, titanium specimens were treated in 5 mol/L NaOH at 100°C for 2 h and then heated in Muffle furnace (200°C for 20 min, 400°C for 20 min, 600°C for 20 min) to generate surfaces with hierarchical microgroove-nanopore topography. The SLA disks were prepared by sandblasting and acid-etching technology. Smooth Ti disks were polished with silicon carbide sandpaper of No. 240, 360, 400, 600, 800, 1000, and 2000 grits in series. All the specimens were washed with acetone, absolute alcohol and MilliQ water sequentially. Finally, specimens were cleaned with double-distilled water (ddH2O) in an ultrasonic cleaner for 30 min, dried at room temperature for 1 h and sterilized in an autoclave at 120°C for 20 min before use.

### Surface Topography Observation

Surface topography of S, SLA, and SLM-AHT surfaces were observed by scanning electron microscopy (SEM, Carl Zeiss SMT Ltd., Cambridge, United Kingdom). High-magnification images were used to qualitatively evaluate the surface nano-scale features. In addition, the average pore diameter was calculated by ImageJ software.

### Cell Culture

Mouse calvaria-derived osteogenic cells MC3T3-E1 from American Type Culture Collection (ATCC) were cultured in fresh DMEM (HyClone, Logan, UT, United States) with 10% FBS (Gibco, New York, NY, United States) and 1% penicillin-streptomycin at 37°C in a humidified atmosphere with 5% CO_2_. Cells were seeded at a density of 1 × 10^4^ cells-well and incubated on specimens. Culture medium was replaced the next day with osteo-induction (OI) medium containing 10% FBS, 1% penicillin-streptomycin, 50 μg-mL ascorbic acid, 10 nmol-L dexamethasone and 5 mmol-L β-glycerophosphate. The medium was changed every 2 days. Samples were cultured for desired times in the following experiments.

### Cell Morphology and Cell Spreading Assay

For cell morphological and spreading analysis, cells were fixed for 30 min at 4°C in 4% paraformaldehyde, subsequently permeabilized with 0.05% Triton X-100 (Sigma-Aldrich) for 10 min. After rinsed with PBS three times, cells were dehydrated through an ethanol series (30%, 50%, 70%, 90%, 95%, and two times 100%), followed by drying in a Critical Point Drier (Balzers CPD 030, Hudson, NH, United States). Characterization of the cell morphology on S, SLA, and SLM-AHT surfaces were carried out by SEM and confocal laser scanning microscope (CLSM, Olympus, Japan).

### Cell Proliferation Assay

For cell proliferation assay, cells were seeded on S, SLA, and SLM-AHT surfaces in culture medium with DAPI (0.5 mg-mL) at a density of 1 × 10^4^ cells-well. After culturing for 6 and 24 h, entire substrates were surveyed photographically at 10× magnification and the DAPI stained nucleus were automatically detected and counted using ImageJ software.

### Scratch Assay

Scratch assay was performed as a model for wounding on S, SLA, and SLM-AHT surfaces. In a confluent monolayer of cells that had been serum-starved in DMEM for 8 h, a scratch was made with a cell scraper. The width of the scratch was measured at the beginning and after 6 or 24 h of culturing in DMEM with 0.5% serum. Cells at the edge of the wound were observed by DAPI staining for cell visualization. Relative closure was calculated by dividing the different group wound closures by that of the blank group.

### Immunofluorescence Staining

MC3T3-E1 were seeded on titanium specimens in 12-well plates at a density of 2 × 10^4^ cells-well. After 3 days of culture, the samples were fixed in 4% paraformaldehyde for 10 min before being permeabilized with 0.05% Triton X-100 (Sigma-Aldrich) for 5 min. The samples were blocked in BSA (5 mg/mL) solution for 1 h. The primary antibody was rabbit anti-Runx2 antibody (1:1000 dilution, Cell Signaling Technology), and cells were incubated overnight at 4°C with it. After incubation, the secondary antibody, a fluorescein isothiocyanate (FITC)-conjugated anti-rabbit antibody (1:2000 dilution, Invitrogen) was applied for 1 h at room temperature. After removing the secondary antibody solution, a FITC-conjugated anti-F-actin antibody (1:200 dilution, Solarbio) was used to stained cytoskeleton for 2 h and then the nucleus were stained with DAPI (5 mg-mL) for 5 min and stored in 1X PBS at 4°C until visualizing with CLSM. All steps of the incubations were performed in a humidified environment at room temperature in the dark. Between each incubation step, the samples were rinsed three times (3 min each) in PBS.

### Immunofluorescence Visualization of FAs

To visualize the FAs, two samples of cells grown on S, SLA, and SLM-AHT surfaces were stained with rabbit anti-Vinculin antibody (1:1000 dilution, Sigma), FITC-conjugated anti-F-actin antibody (1:200 dilution, Solarbio) and DAPI according to manufacture protocols as above described. The samples were analyzed with a Zeiss Axio Imager M2 Optical Microscope (Carl Zeiss, Jena, Germany). High-magnification immunofluorescence imaging was used to study the FAs of the cells.

### Image Analysis

To estimate the number and size of the FAs, the area occupied by vinculin staining was measured and quantified. For each substrate, three individual cells were evaluated after 6 and 24 h from two independent experiments. Immunofluorescence images were taken at 60× to obtain an optimal quality for processing. The captured color images were separated into single-channel greyscale images using the ImageJ split-channel command. To quantify the distribution and size of FAs throughout the cells, punctate vinculin sites were manually traced and the size of each FA was obtained using the Analyze Particle tool in ImageJ. And the FA whose area is larger than 3.14 μm^2^ was defined as mature FA (Parsons et al., 2010; Hanein and Horwitz, 2012). The Shape Descriptors tool in ImageJ was used to measure cell area.

### Western Blot Analysis

The proteins of 6 h, 24 h, 3 days, and 7 days cultured cells on S, SLA, and SLM-AHT surfaces were collected, sonicated and then centrifuged. The proteins in the supernatant were transferred to polyvinylidene difluoride (PVDF) membranes with a semidry transfer apparatus (Bio-Rad, Hercules, CA, United States). The membranes were blocked with 5% dehydrated milk for 2 h and then incubated with following primary antibodies overnight at 4°C. Integrin α2 (Abclonal), Vinculin (Sigma), FAK (Cell Signaling Technology), P-FAK (Cell Signaling Technology), Runx2 (Cell Signaling Technology), Col1a1 (Solarbio), Ocn (Solarbio), PI3K (Cell Signaling Technology), PIP2 (Abcam), PIP3 (Novus), AKT (Cell Signaling Technology), P-AKT (Cell Signaling Technology), and housekeeping protein, glyceraldehyde 3-phosphate dehydrogenase (GAPDH) were used. Membranes were then incubated with a secondary antibody (1:1000 dilution) for 1 h at room temperature, and the antibody-bound proteins were detected using an ECL Western Blotting Analysis System (CWBIO, Beijing, China). The integrated optical density (IOD) was quantified using ImageJ software.

### qRT-PCR Analysis

Quantitative real-time PCR was carried out at 6 h, 24 h, on 3 days and 7 days after cell seeding to evaluate the gene expression levels in cells grown on three titanium surfaces. The oligonucleotide primers for the adhesion and osteogenic related genes integrin α2, Vinculin, Runx2, Col1a1, and Ocn are listed in Table 1. RPO was used as the reference gene. Following incubation, the samples were washed in PBS, and the total RNA was extracted using Trizol reagent (Invitrogen-Life Technologies), according to the manufacturer’s protocols. The amount of total RNA from each sample was quantified using a BioDrop DUO micro-volume spectrophotometer (Montreal Biotech Inc., Canada). qRT-PCR reactions were performed in 10 μl of PCR mixture containing 1 μg of each cDNA sample and specific primers using the QuantiTect SYBR Green PCR Kit (Qiagen, Hilden, Germany). The following conditions were used: 50°C for 10 min, followed by 95°C for 2 min, then 60 cycles of 95°C for 5 s, and 60°C for 10 min.

**TABLE 1:** Primer sequences used for qRT-PCR analysis of gene expression.

Gene	Primer sequence	
Integrin α2	F	AAGTGCCCTGTGGACCTACCCA
	R	TGGTGAGGGTCAATCCCAGGCT
Vinculin	F	ACCTGCAGACCAAAACCAAC
	R	CTTACCGACTCCACGGTCAT
Runx2	F	ATCACTGACGTGCCCAGGCGTA
	R	AGGGCCCAGTTCTGAAGCACCT
Col1a1	F	CTCCTGACGCATGGCCAAGAA
	R	TCAAGCATACCTCGGGTTTCCA
Ocn	F	AGTCTGACAAAGCCTTCA
	R	AAGCAGGGTTAAGCTCACA
RPO	F	TTCATTGTGGGAGCAGAC
	R	CAGCAGTTTCTCCAGAGC

### Construction of Integrin α2 Knockdown Plasmid

To knockdown the integrin α2 (ITGA2) gene, we designed three short hairpin RNAs (shRNA) from https://portals.broadinstitute.org/gpp/public/. The forward and reverse oligonucleotides were synthesized corresponding to the selected shRNA. The shRNA oligonucleotides sequences are listed in Table 2. Mix 1 μl of 10 μM of forward and reverse oligonucleotides in 10 μl of 1X T4 ligase buffer. Incubate at 95°C for 5 min and then ramp down to 25°C at 5°C/min. Prepare a 10 μl ligation reaction mix by adding 50 ng of pLKO.1 digestion product, 1 μl annealed shRNA oligonucleotides, 0.5 μl T4 ligase, 1 μl of 10X T4 ligase buffer. Incubate for 16°C overnight. Transform the ligation product into DH5α cells for 30 min on ice. Heat shock at 42°C for 90 s and return to the ice for 2 min. Add 500 μl of LB medium and incubate at 37°C with shaking for 1 h. Plate the transformation mixture on LB agar plates containing 100 μg-mL ampicillin. Incubate the plates overnight at 37°C in a microbiological incubator. After incubation, pick 3–5 colonies to identify a correct clone for proper insert identification by Sanger sequencing.

**TABLE 2:** Oligonucleotides sequence used for integrin α2 knockdown.

Primer		Oligonucleotides sequence	
shITGA2 1	F	CCGGTCGCAAGAGACT ACGCTTATTCTCGAGAATAAGCGT AGTCTCTTGCGATTTTTG
	R	AATTCAAAAATCGCAAGAGACTA CGCTTATTCTCGAGAATAAGC GTAGTCTCTTGCGA
shITGA2 2	F	CCGGATAGCAGT TCTTGGGTATTTACTCGAGTAAAT ACCCAAGAACTGCTATTTTTTG
	R	AATTCAAAAAATAGCAGTTC TTGGGTATTTACTCGAGTAAATACC CAAGAACTGCTAT
shITGA2 3	F	CCGGGACTGGCTAGTCC AGCGTTTACTCGAGTAAAC GCTGGACTAGCCAGTCTTTTTG
	R	AATTCAAAAAGACTGGCTAGT CCAGCGTTTACTCGAGTAAACGCT GGACTAGCCAGTC

### Construction of Integrin α2 Overexpression Plasmid

To overexpress integrin α2, we divided ITGA2 into A and B two segments and designed 4 clone primers: ITGA2-A-F, ITGA2-A-R, ITGA2-B-F, ITGA2-B-R (Table 3) because of the long sequence. Mix 1 μl of 10 μM forward and reverse oligonucleotides with 1 μl template (MC3T3-E1 cDNA as the template) in 50 μl 1X DNA Polymerase mix. PCR Program are as follows: step1: 95°C Pre-denaturation 3 min; step2: Denaturation 95°C 30 s; step3: Annealing 58°C 30 s; step4: Extension 72°C 90 s; step5: Cycle step2 to step4 35 cycles; step6: Final Extension 72°C 5 min. ITGA2-A and ITGA2-B were amplified. Then the homologous recombination primers oligonucleotides (ITGA2-OE-A-F, ITGA2-OE-B-R) were designed (Table 3). The forward and reverse oligonucleotides were added in the 5′ of ITGA2-A and 3′ of ITGA2-B using the same PCR program, respectively. ITGA2-OE-A and ITGA2-OE-B were amplified. Digest 1 μg pCDH plasmid backbone with 1 μl of EcoRI and 1 μl of BamHI in a final volume of 20 μl of 1 FastDigest Green Buffer. Vazyme Clon Express MultiS One Step Cloning Kit was recombined these 3 fragments. Transformation and identification steps are the same as shRNA cloning.

**TABLE 3:** Oligonucleotides sequence used for integrin α2 overexpression.

Primer		Oligonucleotides sequence	
ITGA2-A	F	ATGGGACCGGGACAGGCAGG
	R	ACCATAGCCATCCAGGGACCTTC
ITGA2-B	F	ACCATAGCCATCCAGGGACCTTC
	R	TTAGCTGTTGAGTTCTGTGGTCTC
ITGA2-OE-A	F	GATGACGATGACAAGGAATTCATGGGA CCGGGACAGGCAGG
ITGA2-OE-B	R	GATCCTTCGCGGCCGCGGATCCTTAGC TGTTGAGTTCTGTGGTCTC

### Virus Infection

To produce recombinant lentivirus, HEK293-T packaging cells were prepared in 10 cm dishes at a density of 70–80%. Then the cells were transfected with the packaging plasmids pVSVG and psPAX8 encoding lentiviral proteins (Gag, Pol, and Env) and the transfer pLKO.1-shRNA or pCDH-ITGA2 plasmid. The medium was changed after 8–12 h. After 24 h, viral supernatants were harvested, and the new medium was added. Viral supernatants were collected the next day. Virus of scramble was a kind of gift from Wu lab. MC3T3-E1 cells with a density of 70% were infected with viral supernatants in the presence of a serum-inactivated medium supplemented. The viral-containing medium was removed after 24 h and cells were grown in serum-containing medium for another day. Cells were then treated with puromycin (2 μg-ml) for selection. The knockdown and overexpression efficiency were confirmed by qRT-PCR and western blot.

### Statistical Analysis

All experiments were repeated at least 3 times to ensure the validity of observations, and all values are expressed as the mean ± standard deviation (SD). The data were tested for homogeneity and then assessed using one-way ANOVA. Error bars represent SD (*n* = 3). *P* < 0.05 was considered significant (^∗^*P* < 0.05, ^∗∗^*P* < 0.01, ^∗∗∗^*P* < 0.005, ^****^P < 0.001).

## Results

### Topography of S, SLA, and SLM-AHT Titanium Surfaces

**FIGURE 1:**
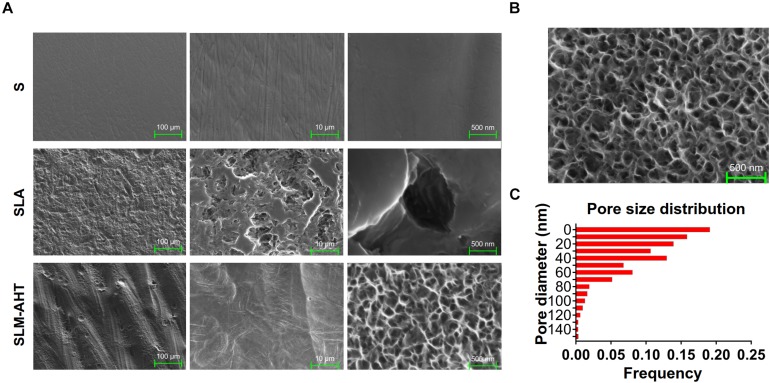
Surface observation of S, SLA and SLM-AHT titanium disks. **(A)** SEM images of the S, SLA and SLM-AHT titanium surfaces. **(B)** SEM images of the hierarchical micro-nano topography titanium surface and the size distribution **(C)** of the nanopores on it.

Topographies of S, SLA and SLM-AHT titanium disks were shown in Figure 1A. As observed by SEM, there exhibited a hierarchical topography combining micro-scale grooves (30–40 um in width) and nano-scale pores (10–100 nm in diameter) on SLM-AHT surface. And irregular micro-scale features with seldom-scattered nano-scale defects could be seen on SLA surface. By contrast, S titanium has a smooth surface without recognizable topographical features. In addition, as shown in Figure 1B, the nanopores on SLM-AHT titanium surface distributed uniformly, most of which were about 40 nm in diameter (Figure 1C).

### Hierarchical Micro-Nano Topography Promoted Cell Adhesion, Proliferation, and Migration

**FIGURE 2:**
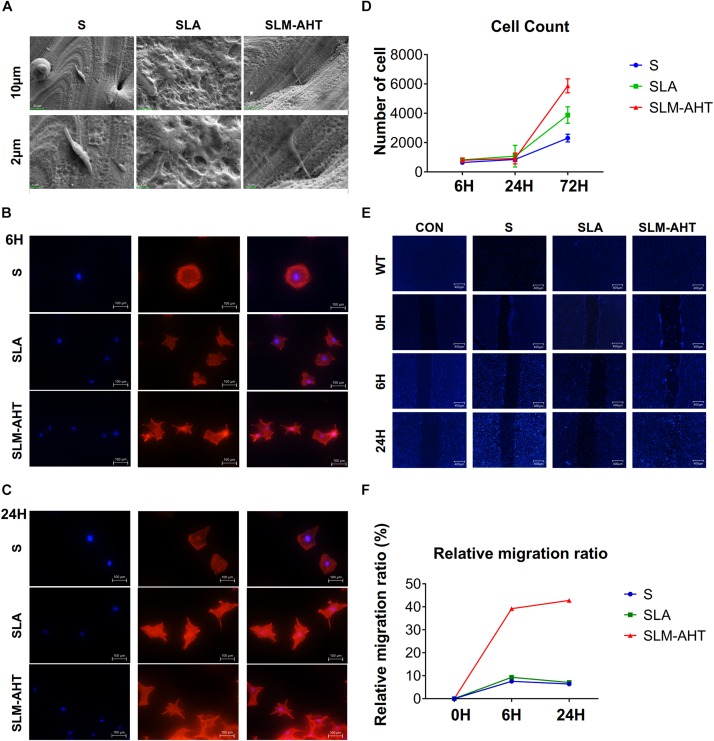
Surface topography influences cell adhesion, proliferation and migration. **(A)** SEM observation of pseudopodia of cell extending on S, SLA, and SLM-AHT titanium surfaces after 6 h of seeding. **(B,C)** Immunofluorescence images of cell (red, F-actin; blue, DAPI) on S, SLA and SLM-AHT titanium surfaces after 6 and 24 h of seeding. **(D)** Cell count on S, SLA and SLM-AHT titanium surfaces for 6, 24, and 72 h. **(E)** Scratch assay of cell on different groups culturing for 0, 6, and 24 h visualized via DAPI (blue) staining. **(F)** Relative closure determined by measuring wound widths from images **(E)** in the upper panel.

To observe cell behaviors on different topography, we seeded MC3T3-E1 cells on S, SLA and SLM-AHT titanium disks, and observed morphology and cell numbers by SEM and CLSM at 6 and 24 h after seeding. As shown in Figure 2A, longer pseudopodia were observed on the SLM-AHT surface (hierarchical micro-nano topography) than S (smooth topography) and SLA (irregular micro-scale topography) surfaces. Immunofluorescence imaging revealed that cells appeared with a round shape and barely any polarity on S surface, while cells exhibited multipolarity on SLA and SLM-AHT surfaces, especially the latter (Figures 2B,C). Significantly increased cell numbers were observed on the hierarchical micro-nano topography compared with the other two surfaces. As shown in Figure 2D, cell numbers on three different surfaces were comparable at 6 and 24 h, but progressive increase in cell number was observed on SLM-AHT surface at 72 h. In the wound healing assay, all the scratches became narrowed somewhat 6 h after scratching, but few cells migrated across the edges of scratches, and there was no obvious difference among the three groups. However, 24 h later, the scratches in SLM-AHT group were completely healed, while those in S and SLA groups were still not (Figures 2E,F), suggesting that SLM-AHT surface could promote cell migration. The rapid migration can establish a cohesive layer of cells on SLM-AHT surface, which is indispensable for cell adhesion and subsequent osteogenic differentiation.

**FIGURE 3:**
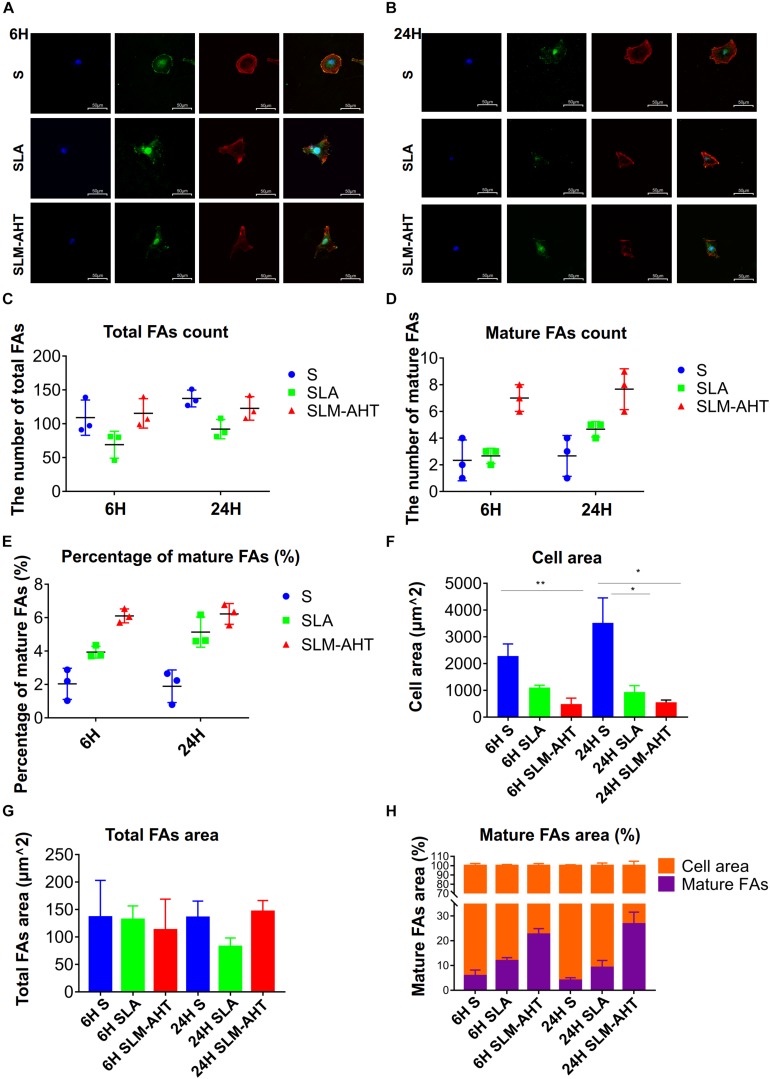
Surface topography influences formation of FAs. **(A,B)** Confocal fluorescence micrographs of cell on three surfaces cultured for 6 **(A)** and 24 h **(B)**. Stained with DAPI (blue) for nuclei, F-actin (red) for actin, and anti-vinculin (green) for vinculin. **(C,D)** The number of total **(C)** and mature **(D)** FAs of MC3T3-E1 cells on three surfaces cultured for 6 and 24 h. **(E)** Percentage of mature FAs number. **(F)** Measurements of the cell area. **(G)** Measurements of the total FAs area. **(H)** Percentage of the cell area occupied by the mature FAs. Statistical significance was determined by one-way ANOVA. Error bars represent SD (*n* = 3). *P < 0.05, **P < 0.01.

To further understand the impact of hierarchical micro-nano topography on cell adhesion, vinculin staining was performed, cells were round on S surface, while appeared polygon in shape on SLA and SLM-AHT surfaces (Figures 3A,B). The size and number of FAs in cells on three surfaces were measured. On average, cells formed more FAs on smooth surface (Figure 3C). However, more mature FAs were found on hierarchical micro-nano topography (Figure 3D), and the percentage of mature FAs on SLM-AHT surface was much higher compared with S and SLA surfaces (Figure 3E). Interestingly, since the quantitative analysis of images revealed that the average cell area on S surface was larger than that of SLA and SLM-AHT surfaces (Figure 3F), while no statistical difference was detected among total FA areas of the 3 groups (Figure 3G), there was a significantly higher proportion of mature FA areas on SLM-AHT surface (Figure 3H).

**FIGURE 4:**
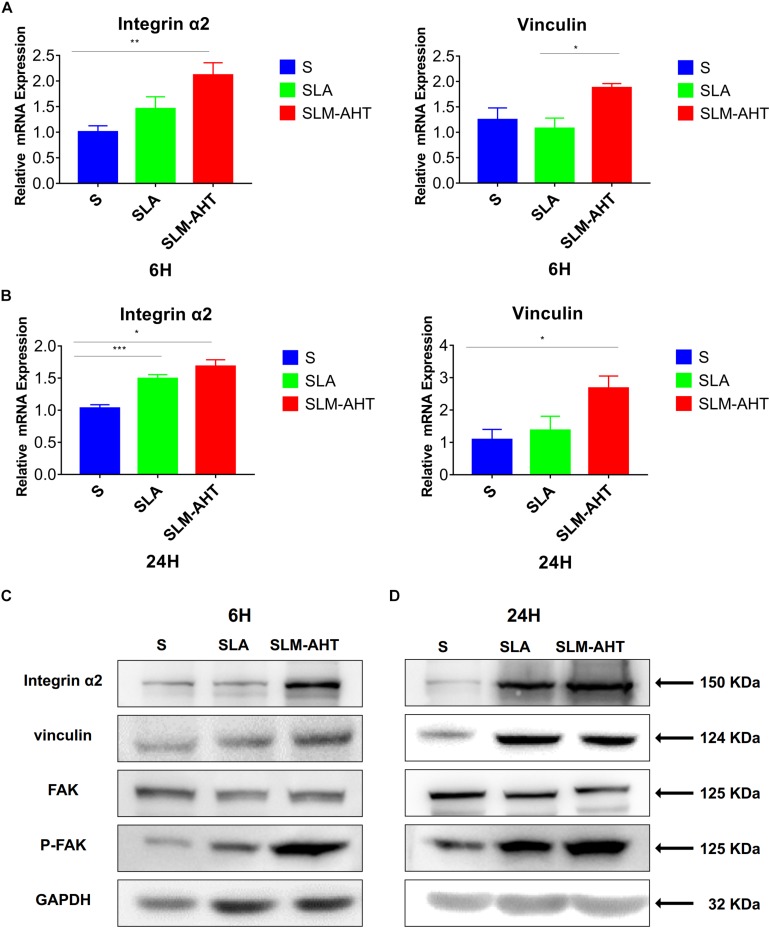
Surface topography influences cell adhesion. **(A,B)** The relative mRNA levels of integrin α2 and vinculin in cell cultured on different titanium surfaces for 6 and 24 h. Statistical significance was determined by one-way ANOVA. Error bars represent SD (*n* = 3). *P < 0.05. **P < 0.01. ***P < 0.005. **(C,D)** Western blot analysis of integrin α2, vinculin, FAK and p-FAK in cell cultured on different titanium surfaces for 6 **(C)** and 24 h **(D)**. Cells were cultured on different materials as indicated above.

Next, we examined the effect of hierarchical micro-nano topography on cell adhesion at molecular level. The expression levels of integrin α2 and vinculin were examined by qRT-PCR and western blot (WB) analysis (Figure 4 and Supplementary Figure 1). Compared with the other two surfaces, SLM-AHT group exhibited a significantly higher mRNA and protein expression level of integrin α2 and vinculin at both 6 and 24 h. Phosphorylated and total FAK protein levels were also evaluated by WB assay (Supplementary Figure 1). Although no significant difference in the total FAK protein expression among three groups was revealed, substantially upregulated FAK phosphorylation level was observed on SLM-AHT surface, suggesting that hierarchical micro-nano topography could promote integrin α2 expression and FAK activation.

### Hierarchical Micro-Nano Topography Promoted Osteogenic Differentiation

**FIGURE 5:**
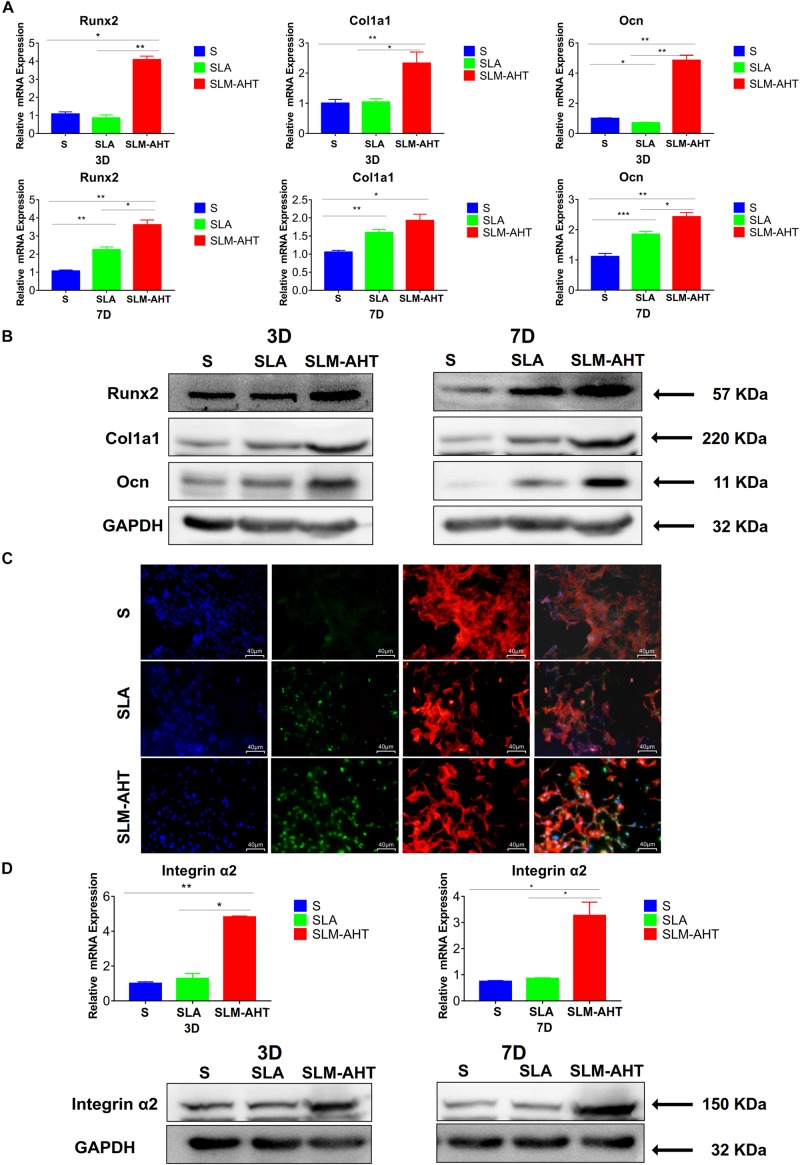
Surface topography influences cell osteogenic differentiation. **(A)** The relative mRNA levels of Runx2, Col1a1 and Ocn in cell cultured on different materials for 3 and 7 days. **(B)** Western blot analysis of Runx2, Col1a1, and Ocn in cell cultured on different materials for 3 and 7 days. **(C)** Immunofluorescence staining showing surface topography dependent diverse expression of Runx2 in cell cultured on different materials for 3 days. (red, F-actin; blue, DAPI; green, Runx2). **(D)** The relative mRNA and protein levels of integrin α2 in cell cultured on different materials for 3 and 7 days. Statistical significance was determined by one-way ANOVA. Error bars represent SD (*n* = 3). *P < 0.05, **P < 0.01, ***P < 0.005.

As shown in Figures 5A,B and Supplementary Figure 2A, the expression levels of Runx2, Col1a1, and Ocn in cells cultured on SLM-AHT surface were significantly higher than those of S and SLA groups on both day 3 and day 7. In addition, immunofluorescence staining further confirmed that Runx2 expression levels were significantly increased in cells cultured on SLM-AHT surface (Figure 5C), indicating an obvious osteogenic promoting effect of the hierarchical micro-nano topography. Furthermore, the expression levels of integrin α2 in cells cultured on SLM-AHT surface were also found significantly upregulated compared to S and SLA groups, while there was no significant difference between the latter two groups (Figure 5D and Supplementary Figure 2B), suggesting integrin α2 was involved in hierarchical micro-nano topography regulating osteogenic differentiation.

### The Role of α2 -PI3K-AKT Signaling Axis

**FIGURE 6:**
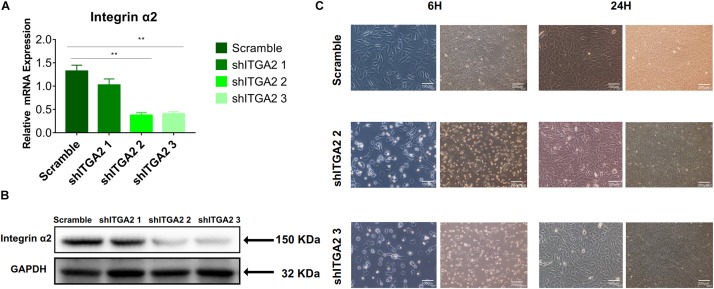
Efficiency of integrin α2 knockdown. **(A)** The relative mRNA expression levels of integrin α2 in scramble and integrin α2 silenced cell for 3 days. Statistical significance was determined by one-way ANOVA. Error bars represent SD (*n* = 3). **P < 0.01. **(B)** Western blot analysis of integrin α2 in scramble and integrin α2 silenced cell for 3 days. **(C)** Microscope images of morphology of scramble and integrin α2 silenced cell for 6 and 24 h.

**FIGURE 7:**
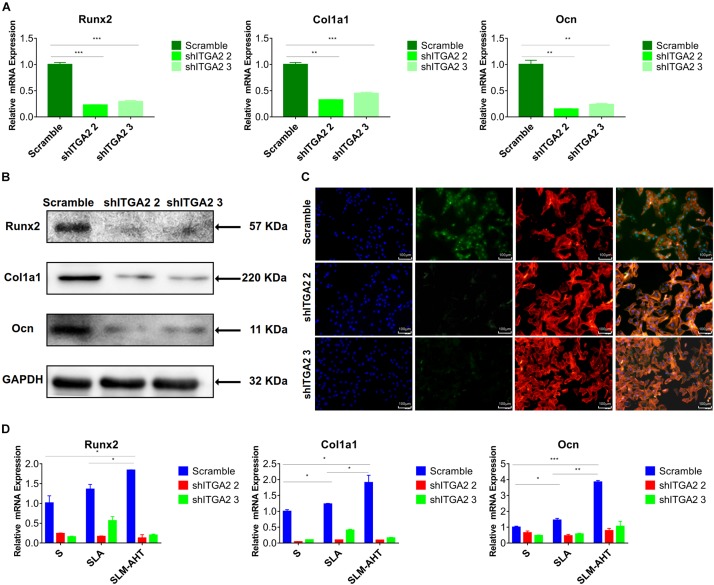
Integrin α2 influences cell osteogenic differentiation. **(A)** The relative mRNA levels of Runx2, Col1a1, and Ocn in scramble and integrin α2 silenced cell. **(B)** Western blot analysis of Runx2, Col1a1, and Ocn proteins in scramble and integrin α2 silenced cell. **(C)** Immunofluorescence staining showing integrin α2 dependent diverse expression of Runx2 (red, F-actin; blue, DAPI: green, Runx2). **(D)** The relative mRNA levels of Runx2, Col1a1, and Ocn in scramble and integrin α2 silenced cell culturing on S, SLA, and SLM-AHT surfaces for 3 days. Statistical significance was determined by one-way ANOVA. Error bars represent SD (*n* = 3). *P < 0.05, **P < 0.01, ***P < 0.005.

To further investigate the role of integrin α2 and its relationship with PI3K-AKT signaling, we designed three shRNAs. Two of them (shITGA2 2 and shITGA2 3) were proved effective and with no adverse effect on cell morphology as well as cell proliferation (Figures 6A–C and Supplementary Figure 3). Accordingly, scramble, shITGA2 2 and shITGA2 3 cells were seeded on both cell-culture dishes and three titanium specimens to study the role of integrin α2 in osteogenic differentiation. qRT-PCR and WB analysis revealed that knockdown of integrin α2 dramatically decreased expression of osteogenic markers including Runx2, Col1a1 and Ocn (Figures 7A,B and Supplementary Figure 4). And the significantly downregulated expression of Runx2 was further confirmed by immunofluorescence staining (Figure 7C). Noticeably, scramble cells cultured on SLM-AHT surface exhibited significantly increased expression of Runx2, Col1a1 and Ocn compared to S and SLA surface, while integrin α2 silenced cells showed no difference of osteogenic gene expression on three surfaces (Figure 7D), indicating that integrin α2 played a key role in hierarchical micro-nano topography directing osteogenic differentiation.

**FIGURE 8:**
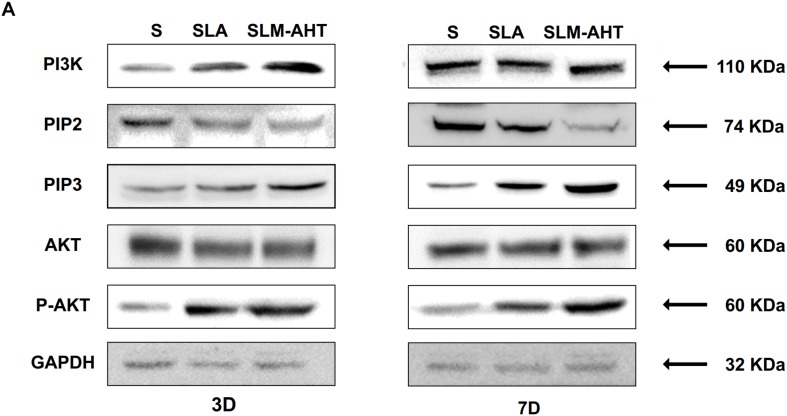
Surface topography influences PI3K-AKT signaling pathway. **(A,B)** Western blot analysis of PI3K, PIP2, PIP3, AKT, and p-AKT in cell cultured on S, SLA and SLM-AHT surfaces for 3 and 7 days. Cells were cultured on different materials as indicated above.

**FIGURE 9:**
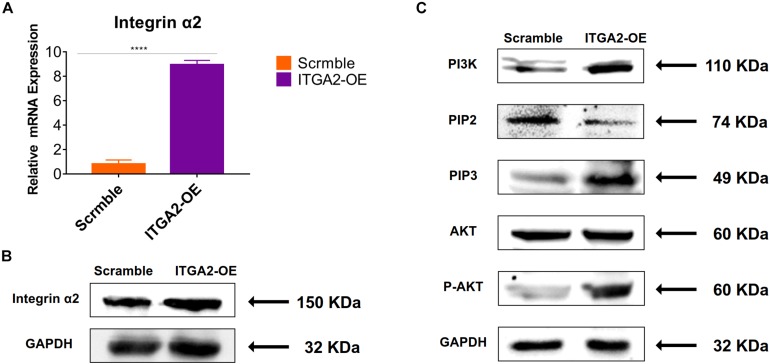
Integrin α2 overexpression activates PI3K-AKT signal pathway. **(A)** The relative mRNA levels of integrin α2 in scramble and integrin α2 overexpressed cell for 3 days. Statistical significance was determined by one-way ANOVA. Error bars represent SD (*n* = 3). ****P < 0.001. **(B)** Western blot analysis of integrin α2 in scramble and integrin α2 overexpressed cell. **(C)** Western blot analysis of PI3K, PIP2, PIP3, AKT, and p-AKT in scramble and integrin α2 overexpressed cell.

**FIGURE 10:**
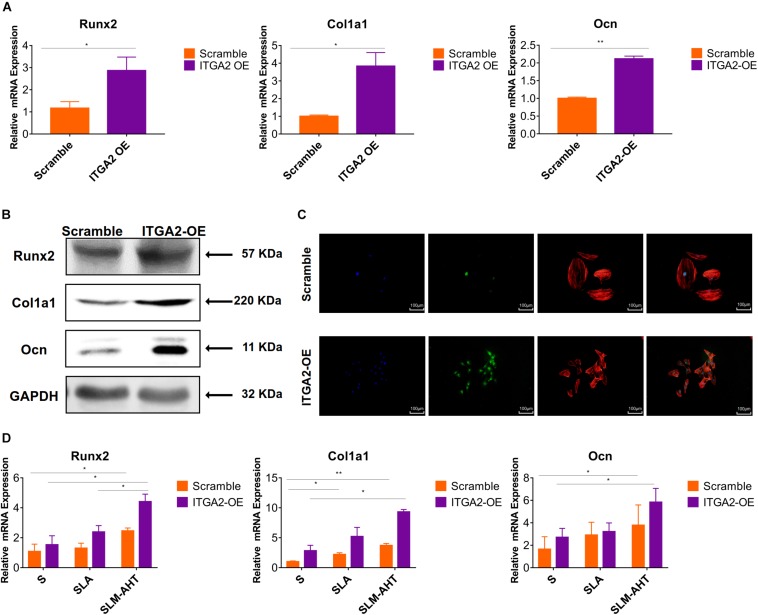
Integrin α2 overexpression enhances cell osteogenic differentiation. **(A)** The relative mRNA levels of Runx2, Col1a1, and Ocn in scramble and integrin α2 overexpressed cell for 3 days. **(B)** Western blot analysis of Runx2, Col1a1, and Ocn proteins in scramble and integrin α2 overexpressed cell for 3 days. **(C)** Immunofluorescence staining showing integrin α2 dependent diverse expression of Runx2 (red, F-actin; blue, DAPI: green, Runx2). **(D)** The relative mRNA levels of Runx2, Col1a1, and Ocn in scramble and integrin α2 overexpressed cell cultured on S, SLA and SLM-AHT surfaces for 3 days. Statistical significance was determined by one-way ANOVA. Error bars represent SD (*n* = 3). *P < 0.05, **P < 0.01.

The activity of PI3K-AKT signaling was then investigated on different surfaces. The related factors including PI3K, PIP2, PIP3, total AKT and phosphorylated AKT (P-AKT) were detected by WB. As shown in Figure 8 and Supplementary Figure 5, the protein expression of PI3K and PIP3 was higher on SLM-AHT surface than S and SLA surfaces on both day 3 and 7, while PIP2 had a lower expression on SLM-AHT surface compared to the other two groups. Interestingly, although no marked alteration in total AKT expression on all three surfaces was found, significantly increased AKT phosphorylation level was revealed on SLM-AHT surface, confirming the activation of PI3K-AKT signaling on the hierarchical micro-nano topography. We then examined the interaction of integrin α2 and PI3K-AKT signaling. Integrin α2 was stably overexpressed in MC3T3-E1 cells (ITGA2-OE) (Figures 9A,B and Supplementary Figure 6A). WB analysis showed that integrin α2 overexpression significantly promoted expression levels of PI3K and PIP3, while strongly decreased PIP2 expression (Figure 9C and Supplementary Figure 6B). Furthermore, while total AKT expression did not change significantly, increased P-AKT was observed after integrin α2 overexpression. The above results suggested that PI3K-AKT signaling was highly influenced by integrin α2. That was to say, there exists an integrin α2-PI3K-AKT signaling axis which was possibly involved in the regulation of cell behaviors including osteogenic differentiation. In addition, scramble and forced integrin α2 cells were seeded on both cell-culture dishes and three titanium specimens. And it was found that the mRNA and protein expression levels of osteogenic markers were increased after integrin α2 overexpression (Figures 10A,B and Supplementary Figure 7). Furthermore, the significantly upregulated expression of Runx2 was observed by immunofluorescence staining after integrin α2 overexpression (Figure 10C). Noticeably, both integrin α2 overexpressed cells and scramble cells cultured on SLM-AHT surface exhibited significantly increased mRNA expression of Runx2, Col1a1 and Ocn compared to S and SLA groups (Figure 10D), once again proving the key role of integrin α2 in surface topography regulating cell fate.

## Discussion

In the human body, cells are exposed to complex microenvironments consisting of varying micro-scale and nano-scale structural features which convey different topographical cues to regulate cell behaviors (Fu et al., 2020). It is believed that the development of appropriate hierarchical micro-nano topographies mimicking the structure of natural bone helps to improve the osseointegration ability of intraosseous implants (Gongadze et al., 2011; Khang et al., 2012; Xu et al., 2016; Shah et al., 2018). And recent studies revealed that surface with nano-scale features was effective at improving osteoblasts adhesion and differentiation (Gautrot et al., 2014; Rosa et al., 2014; Huang et al., 2016; Guadarrama Bello et al., 2017; Lopes et al., 2019). However, the mechanism by which surface topography manipulates cell fate remains controversial. In this study, hierarchical micro-nano topography (SLM-AHT) with micro-scale grooves and nano-scale pores was fabricated and compared with smooth topography (S) as well as irregular micro-scale topography (SLA) surfaces to investigate the mechanism involved in cell-surface interactions.

Topography-induced changes in cell morphology could be conveniently observed at the initial stage of cell-surface contact (Dupont, 2016) and would greatly influence subsequent cell behaviors (Nasrollahi et al., 2016). In this study, cells showed multipolarity on SLM-AHT surface while showed round on the other surfaces, which could be attributed to the large amount of sharp convex or spikes of hierarchical micro-nano topography. These features have the highest negative surface charge density (Gongadze et al., 2011), and could, therefore, recruit more positively charged anchor proteins to improve cell adhesion (Smeets et al., 2009), as observed in the following FA visualization and detection of integrin α2, vinculin and p-FAK expression.

Focal adhesions were intensively studied because they are regarded as important transducers of mechanical cues including topography (You et al., 2014). In this study, more FAs were found in cells cultured on S surface. However, most of them are dot-like nascent adhesions (NAs) or premature round focal complexes (FCs), which are not stable and usually disappear within several minutes (Valdembri and Serini, 2012). Since total FAs cannot present the real adhesion status of cells, mature FAs were measured. And cells cultured on SLM-AHT surface exhibited much more mature FAs, indicating hierarchical micro-nano topography may contribute to cell adhesion via enhancing the maturation of FAs. The possible reason may be that FAs are favorably constructed with an average integrin interspacing of about 45 nm (Dalby et al., 2014), while the diameter of nanopores on SLM-AHT surface is about 40 nm. The evenly distributed nano-scale features on the surface substantially match the preferable interspacing of integrins to form mature FAs, and therefore improve cell adhesion (Cavalcanti-Adam et al., 2007).

In consistence with previous studies, our results showed that the titanium surface with hierarchical microgroove-nanopore topography would favor osteogenic differentiation. More importantly, the role of integrin α2, an important component of FAs, in the observed topography induced osteogenic differentiation was thoroughly investigated in this study. Compared to S and SLA surfaces, protein expression level of integrin α2 was found significantly increased at early stage of cell cultured on SLM-AHT surface, and concomitantly, cells on SLM-AHT surface also exhibited an early increase in mRNA expression levels of osteogenic markers. Moreover, we established the causal-effect relationship through gain and loss of function of integrin α2 in cells cultured on SLM-AHT. Although the osteogenic marker expression was higher on SLA surface than on the S surface on day 7, the protein expression level of integrin α2 showed no significant difference between the two groups, indicating that less prominent osteogenic promoting effect of irregular micro-scale topography was not integrin α2 dependent (Huang et al., 2019). Therefore, integrin α2 mediated osteogenic differentiation could be considered topography specific, and in the existing three surfaces, only hierarchical micro-nano topography (SLM-AHT) could activate this process. And our finding helps to endorse the recently proposed possible function of integrin α2 in bone formation (Leem et al., 2016; Raines et al., 2019).

Moreover, the downstream signaling of integrin α2 in promoting osteogenic differentiation was further investigated in this study. Our outcomes showed that PI3K-AKT signaling pathway was activated on SLM-AHT surface, combing with high expression of integrin α2. Since PI3K-AKT signaling had been proved involved in multiple cell functions including cell proliferation, apoptosis, growth, glucose metabolism, migration and differentiation (Gu et al., 2013; Choi et al., 2014; Xu et al., 2015; Fan et al., 2018), we supposed that there might be cross-talk between integrin α2 and PI3K-AKT signaling pathway, and hierarchical micro-nano topography by itself could affect this integrin α2-PI3K-AKT signaling axis. However, whether integrin α2 could activate PI3K-AKT signaling pathway was still unknown. Thus, we stably upregulated endogenous integrin α2 expression to investigate the effect of integrin α2 on PI3K-AKT signaling pathway. To our delight, the high expression level of integrin α2 was indeed accompanied by an activated PI3K-AKT signaling. Furthermore, following by PI3K-AKT signaling pathway activation, osteogenic markers were also upregulated in integrin α2 overexpressed cells. Altogether, it could be inferred that forced expression of integrin α2 could activate PI3K-AKT signaling pathway and thereby promote osteogenic differentiation.

To our knowledge, this is the very first report illustrating that osteogenic differentiation induced by hierarchical micro-nano topography is mediated by activation of integrin α2-PI3K-AKT signaling axis, as well as is the first study demonstrating overexpression of integrin α2 is sufficient to activate PI3K-AKT signaling pathway on titanium surfaces. Our research contributes to surface modification targeting the enhancement of osteogenic capacity. And for cells with compromised osteogenic capacity, it provides a possibility of overexpressing integrin α2 to promote the osteogenic differentiation. However, integrin α2 is also involved in metabolism of tumor cells (Adorno-Cruz and Liu, 2019). Thus, the safety of long-time high expression of integrin α2 is worrisome which needs rigorous evaluation. Despite a helpful step in uncovering a novel signaling axis involved in cell-surface interactions, our understanding of it remains incomplete and there are some shortcomings in this study. Whether the same mechanism can play its role in vivo, and whether integrin α2 can activate other signaling pathways to promote osteogenic differentiation of cell cultured on surface with hierarchical micro-nano topography, are still unknown. Collectively, we proposed α2-PI3K-AKT signaling axis plays a crucial role in hierarchical micro-nano topography induced osteogenic differentiation. More mechanistic insights still require further studies.

## Data Availability Statement

The original contributions presented in the study are included in the article/Supplementary Material, further inquiries can be directed to the corresponding authors.

## Author Contributions

All the authors were involved in this work. XW and LS conceived the idea of the study. YT fabricated and characterized the materials. HZ and XX designed and performed the experiments. QG and FD helped with the experiments and provided constructive discussions. YY provided the financially supporting for this work. ZF analyzed the data. HZ, XW, and LS interpreted the data and wrote the manuscript.

## Conflict of Interest

The authors declare that the research was conducted in the absence of any commercial or financial relationships that could be construed as a potential conflict of interest.

## References

[B1] Adorno-Cruz V. Liu H. (2019). Regulation and functions of integrin alpha2 in cell adhesion and disease. *Genes Dis.* 6 16–24. 10.1016/j.gendis.2018.12.003 30906828 PMC6411621

[B2] Anselme K. Davidson P. Popa A. M. Giazzon M. Liley M. Ploux L. (2010). The interaction of cells and bacteria with surfaces structured at the nanometre scale. *Acta Biomater.* 6 3824–3846. 10.1016/j.actbio.2010.04.001 20371386

[B3] Arienti C. Pignatta S. Tesei A. (2019). Epidermal growth factor receptor family and its role in gastric cancer. *Front. Oncol.* 9:1308 10.3389/fonc.2019.01308 PMC6901979 31850207

[B4] Bachir A. I. Zareno J. Moissoglu K. Plow E. F. Gratton E. Horwitz A. R. (2014). Integrin-associated complexes form hierarchically with variable stoichiometry in nascent adhesions. *Curr. Biol.* 24 1845–1853. 10.1016/j.cub.2014.07.011 25088556 PMC4138543

[B5] Cavalcanti-Adam E. A. Volberg T. Micoulet A. Kessler H. Geiger B. Spatz J. P. (2007). Cell spreading and focal adhesion dynamics are regulated by spacing of integrin ligands. *Biophys. J.* 92 2964–2974. 10.1529/biophysj.106.089730 17277192 PMC1831685

[B6] Chen W. Shao Y. Li X. Zhao G. Fu J. (2014). Nanotopographical surfaces for stem cell fate control: engineering mechanobiology from the bottom. *Nano Today* 9 759–784. 10.1016/j.nantod.2014.12.002 25883674 PMC4394389

[B7] Choi Y. H. Kim Y. J. Jeong H. M. Jin Y. H. Yeo C. Y. Lee K. Y. (2014). Akt enhances Runx2 protein stability by regulating Smurf2 function during osteoblast differentiation. *FEBS J.* 281 3656–3666. 10.1111/febs.12887 24961731

[B8] Chuang L. S. H. Ito K. Ito Y. (2013). RUNX family: regulation and diversification of roles through interacting proteins. *Int. J. Cancer* 132 1260–1271. 10.1002/ijc.27964 23180629

[B9] Cimmino C. Rossano L. Netti P. A. Ventre M. (2018). Spatio-temporal control of cell adhesion: toward programmable platforms to manipulate cell functions and fate. *Front. Bioeng. Biotechnol.* 6:190 10.3389/fbioe.2018.00190 PMC6288377 30564573

[B10] Cui Y. Zhu T. Li D. Li Z. Leng Y. Ji X. (2019). Bisphosphonate-functionalized scaffolds for enhanced bone regeneration. *Adv. Healthc. Mater.* 8:e1901073. 10.1002/adhm.201901073 31693315

[B11] Dalby M. J. Gadegaard N. Oreffo R. O. (2014). Harnessing nanotopography and integrin-matrix interactions to influence stem cell fate. *Nat. Mater.* 13 558–569. 10.1038/nmat3980 24845995

[B12] De Franceschi N. Hamidi H. Alanko J. Sahgal P. Ivaska J. (2015). Integrin traffic – The update. *J. Cell Sci.* 128 839–852. 10.1242/jcs.161653 25663697 PMC4342575

[B13] Denchai A. Tartarini D. Mele E. (2018). Cellular response to surface morphology: electrospinning and computational modeling. *Front. Bioeng. Biotechnol.* 6:155 10.3389/fbioe.2018.00155 PMC6207584 30406098

[B14] Deng C. Lin R. Zhang M. Qin C. Yao Q. Wang L. (2019). Micro/nanometer-structured scaffolds for regeneration of both cartilage and subchondral bone. *Adv. Funct. Mater.* 29:1806068.

[B15] Dupont S. (2016). Role of YAP/TAZ in cell-matrix adhesion-mediated signalling and mechanotransduction. *Exp. Cell Res.* 343 42–53. 10.1016/j.yexcr.2015.10.034 26524510

[B16] Edlind M. P. Hsieh A. C. (2014). PI3K-AKT-mTOR signaling in prostate cancer progression and androgen deprivation therapy resistance. *Asian J. Androl.* 16 378–386. 24759575 10.4103/1008-682X.122876 PMC4023363

[B17] Fan Y. S. Li Q. Hamdan N. Bian Y. F. Zhuang S. Fan K. (2018). Tetrahydroxystilbene glucoside regulates proliferation, differentiation, and OPG/RANKL/M-CSF expression in MC3T3-E1 cells via the PI3K/Akt pathway. *Molecules* 23:E2306. 10.3390/molecules23092306 PMC6225160 30201908

[B18] Feng S. Zhou L. Zhang Y. Lu S. Long M. (2018). Mechanochemical modeling of neutrophil migration based on four signaling layers, integrin dynamics, and substrate stiffness. *Biomech. Model. Mechanobiol.* 17 1611–1630. 10.1007/s10237-018-1047-2 29968162

[B19] Fu J. Liu X. Tan L. Cui Z. Liang Y. Li Z. (2020). Modulation of the mechanosensing of mesenchymal stem cells by laser-induced patterning for the acceleration of tissue reconstruction through the Wnt/beta-catenin signaling pathway activation. *Acta Biomater.* 101 152–167. 10.1016/j.actbio.2019.10.041 31678738

[B20] Gautrot J. E. Malmstrom J. Sundh M. Margadant C. Sonnenberg A. Sutherland D. S. (2014). The nanoscale geometrical maturation of focal adhesions controls stem cell differentiation and mechanotransduction. *Nano Lett.* 14 3945–3952. 10.1021/nl501248y 24848978

[B21] Geiger B. Spatz J. P. Bershadsky A. D. (2009). Environmental sensing through focal adhesions. *Nat. Rev. Mol. Cell Biol.* 10 21–33. 10.1038/nrm2593 19197329

[B22] Ginsberg M. H. (2014). Integrin activation. *BMB Rep.* 47 655–659. 25388208 10.5483/BMBRep.2014.47.12.241 PMC4345508

[B23] Gongadze E. Kabaso D. Bauer S. Slivnik T. Schmuki P. van Rienen U. (2011). Adhesion of osteoblasts to a nanorough titanium implant surface. *Int. J. Nanom.* 6 1801–1816. 10.2147/IJN.S21755 PMC3173045 21931478

[B24] Gorelik R. Gautreau A. (2014). Quantitative and unbiased analysis of directional persistence in cell migration. *Nat. Protoc.* 9 1931–1943. 10.1038/nprot.2014.131 25033209

[B25] Gu Y. X. Du J. Si M. S. Mo J. J. Qiao S. C. Lai H. C. (2013). The roles of PI3K/Akt signaling pathway in regulating MC3T3-E1 preosteoblast proliferation and differentiation on SLA and SLActive titanium surfaces. *J. Biomed. Mater. Res. A* 101 748–754. 10.1002/jbm.a.34377 22941963

[B26] Guadarrama Bello D. Fouillen A. Badia A. Nanci A. (2017). A nanoporous titanium surface promotes the maturation of focal adhesions and formation of filopodia with distinctive nanoscale protrusions by osteogenic cells. *Acta Biomater.* 60 339–349. 10.1016/j.actbio.2017.07.022 28728969

[B27] Hanein D. Horwitz A. R. (2012). The structure of cell-matrix adhesions: the new frontier. *Curr. Opin. Cell Biol.* 24 134–140. 10.1016/j.ceb.2011.12.001 22196929 PMC3294145

[B28] Huang Q. Elkhooly T. A. Liu X. Zhang R. Yang X. Shen Z. (2016). Effects of hierarchical micro/nano-topographies on the morphology, proliferation and differentiation of osteoblast-like cells. *Colloids Surf. B Biointerfaces* 145 37–45. 10.1016/j.colsurfb.2016.04.031 27137801

[B29] Huang T. B. Li Y. Z. Yu K. Yu Z. Wang Y. Jiang Z. W. (2019). Effect of the Wnt signal-RANKL/OPG axis on the enhanced osteogenic integration of a lithium incorporated surface. *Biomater. Sci.* 7 1101–1116. 10.1039/c8bm01411f 30633253

[B30] Hui-Min Hu L. Y. Wang Z. Liu Y.-W. Fan J.-Z. Fan J. (2013). Overexpression of integrin a2 promotes osteogenic differentiation of hBMSCs from senile osteoporosis through the ERK pathway. *Int. J. Clin. Exp. Pathol.* 5 841–852. PMC3638094 23638215

[B31] Izard T. Brown D. T. (2016). Mechanisms and functions of vinculin interactions with phospholipids at cell adhesion sites. *J. Biol. Chem.* 291 2548–2555. 10.1074/jbc.r115.686493 26728462 PMC4742724

[B32] Janssen S. Gach S. Kant S. Aveic S. Rutten S. Olschok S. (2020). Enhanced osteogenic differentiation of human mesenchymal stromal cells as response to periodical microstructured Ti6Al4V surfaces. *J. Biomed. Mater. Res. B Appl. Biomater.* 108, 2218–2226. 31981406 10.1002/jbm.b.34559

[B33] Karimi F. O’Connor A. J. Qiao G. G. Heath D. E. (2018). Integrin clustering matters: a review of biomaterials functionalized with multivalent integrin-binding ligands to improve cell adhesion, migration, differentiation, angiogenesis, and biomedical device integration. *Adv. Healthc. Mater.* 7:e1701324. 10.1002/adhm.201701324 29577678

[B34] Karsenty G. Kronenberg H. M. Settembre C. (2009). Genetic control of bone formation. *Annu. Rev. Cell Dev. Biol.* 25 629–648. 10.1146/annurev.cellbio.042308.113308 19575648

[B35] Khang D. Choi J. Im Y. M. Kim Y. J. Jang J. H. Kang S. S. (2012). Role of subnano-, nano- and submicron-surface features on osteoblast differentiation of bone marrow mesenchymal stem cells. *Biomaterials* 33 5997–6007. 10.1016/j.biomaterials.2012.05.005 22632766

[B36] Kim J. Kim H. N. Lim K. T. Kim Y. Seonwoo H. Park S. H. (2013). Designing nanotopographical density of extracellular matrix for controlled morphology and function of human mesenchymal stem cells. *Sci. Rep.* 3:3552. 10.1038/srep03552 PMC6506445 24352057

[B37] Leem Y. H. Lee K. S. Kim J. H. Seok H. K. Chang J. S. Lee D. H. (2016). Magnesium ions facilitate integrin alpha 2- and alpha 3-mediated proliferation and enhance alkaline phosphatase expression and activity in hBMSCs. *J. Tissue Eng. Regen. Med.* 10 E527–E536. 24616281 10.1002/term.1861

[B38] Li J. Yu Y. Myungwoong K. Li K. Mikhail J. Zhang L. (2017). Manipulation of cell adhesion and dynamics using RGD functionalized polymers. *J. Mater. Chem. B* 5 6307–6316. 10.1039/c7tb01209h 32264447

[B39] Li N.-B. Xiao G.-Y. Liu B. Wang Z. Zhu R.-F. Lu Y.-P. (2016). Rapid deposition of spherical apatite on alkali–heat treated titanium in modified simulated body fluid at high temperature. *Surf. Coat. Technol.* 301 121–125. 10.1016/j.surfcoat.2015.12.067

[B40] Lopes H. B. Freitas G. P. Fantacini D. M. C. Picanco-Castro V. Covas D. T. Rosa A. L. (2019). Titanium with nanotopography induces osteoblast differentiation through regulation of integrin alphaV. *J. Cell. Biochem.* 120 16723–16732. 10.1002/jcb.28930 31090958

[B41] Lou H. Y. Zhao W. Li X. Duan L. Powers A. Akamatsu M. (2019). Membrane curvature underlies actin reorganization in response to nanoscale surface topography. *Proc. Natl. Acad. Sci. U.S.A.* 116 23143–23151. 10.1073/pnas.1910166116 31591250 PMC6859365

[B42] Mattila P. K. Lappalainen P. (2008). Filopodia: molecular architecture and cellular functions. *Nat. Rev. Mol. Cell Biol.* 9 446–454. 10.1038/nrm2406 18464790

[B43] Michael M. Parsons M. (2020). New perspectives on integrin-dependent adhesions. *Curr. Opin. Cell Biol.* 63 31–37. 10.1016/j.ceb.2019.12.008 31945690 PMC7262580

[B44] Nasrollahi S. Banerjee S. Qayum B. Banerjee P. Pathak A. (2016). Nanoscale matrix topography influences microscale cell motility through adhesions, actin organization, and cell shape. *ACS Biomater. Sci. Eng.* 3 2980–2986. 10.1021/acsbiomaterials.6b00554 33418718

[B45] Olivares-Navarrete R. Rodil S. E. Hyzy S. L. Dunn G. R. Almaguer-Flores A. Schwartz Z. (2015). Role of integrin subunits in mesenchymal stem cell differentiation and osteoblast maturation on graphitic carbon-coated microstructured surfaces. *Biomaterials* 51 69–79. 10.1016/j.biomaterials.2015.01.035 25770999 PMC4636027

[B46] Parsons J. T. Horwitz A. R. Schwartz M. A. (2010). Cell adhesion: integrating cytoskeletal dynamics and cellular tension. *Nat. Rev. Mol. Cell Biol.* 11 633–643. 10.1038/nrm2957 20729930 PMC2992881

[B47] Raines A. L. Berger M. B. Schwartz Z. Boyan B. D. (2019). Osteoblasts grown on microroughened titanium surfaces regulate angiogenic growth factor production through specific integrin receptors. *Acta Biomater.* 97 578–586. 10.1016/j.actbio.2019.07.036 31349056 PMC7250132

[B48] Robling A. G. Castillo A. B. Turner C. H. (2006). Biomechanical and molecular regulation of bone remodeling. *Annu. Rev. Biomed. Eng.* 8 455–498. 10.1146/annurev.bioeng.8.061505.095721 16834564

[B49] Rosa A. L. Kato R. B. Castro Raucci L. M. Teixeira L. N. de Oliveira F. S. Bellesini L. S. (2014). Nanotopography drives stem cell fate toward osteoblast differentiation through alpha1beta1 integrin signaling pathway. *J. Cell. Biochem.* 115 540–548. 10.1002/jcb.24688 24122940

[B50] Saruta J. Sato N. Ishijima M. Okubo T. Hirota M. Ogawa T. (2019). Disproportionate effect of sub-micron topography on osteoconductive capability of titanium. *Int. J. Mol. Sci.* 20:E4027. 10.3390/ijms20164027 PMC6720784 31426563

[B51] Shah F. A. Thomsen P. Palmquist A. (2018). A review of the impact of implant biomaterials on osteocytes. *J. Dent. Res.* 97 977–986. 10.1177/0022034518778033 29863948 PMC6055115

[B52] Siebers M. C. ter Brugge P. J. Walboomers X. F. Jansen J. A. (2005). Integrins as linker proteins between osteoblasts and bone replacing materials. A critical review. *Biomaterials* 26 137–146. 10.1016/j.biomaterials.2004.02.021 15207460

[B53] Skoog S. A. Kumar G. Narayan R. J. Goering P. L. (2018). Biological responses to immobilized microscale and nanoscale surface topographies. *Pharmacol. Ther.* 182 33–55. 10.1016/j.pharmthera.2017.07.009 28720431

[B54] Smeets R. Kolk A. Gerressen M. Driemel O. Maciejewski O. Hermanns-Sachweh B. (2009). A new biphasic osteoinductive calcium composite material with a negative Zeta potential for bone augmentation. *Head Face Med.* 5:13. 10.1186/1746-160X-5-13 PMC2706807 19523239

[B55] Sun Z. Guo S. S. Fassler R. (2016). Integrin-mediated mechanotransduction. *J. Cell Biol.* 215 445–456. 10.1083/jcb.201609037 27872252 PMC5119943

[B56] Sun Z. Lambacher A. Fässler R. (2014). Nascent adhesions: from fluctuations to a hierarchical organization. *Curr. Biol.* 24 R801–R803. 25202871 10.1016/j.cub.2014.07.061

[B57] Valdembri D. Serini G. (2012). Regulation of adhesion site dynamics by integrin traffic. *Curr. Opin. Cell Biol.* 24 582–591. 10.1016/j.ceb.2012.08.004 22981739

[B58] Wozniak M. A. Modzelewska K. Kwong L. Keely P. J. (2004). Focal adhesion regulation of cell behavior. *Biochim. Biophys. Acta* 1692 103–119. 15246682 10.1016/j.bbamcr.2004.04.007

[B59] Xu J. Y. Chen X. S. Zhang C. Y. Liu Y. Wang J. Deng F. L. (2016). Improved bioactivity of selective laser melting titanium: surface modification with micro-/nano-textured hierarchical topography and bone regeneration performance evaluation. *Mater. Sci. Eng. C Mater. Biol. Appl.* 68 229–240. 10.1016/j.msec.2016.05.096 27524017

[B60] Xu W. Yang Z. Lu N. (2015). A new role for the PI3K/Akt signaling pathway in the epithelial-mesenchymal transition. *Cell Adhes. Migr.* 9 317–324. 10.1080/19336918.2015.1016686 PMC4594353 26241004

[B61] Yoon S. O. Shin S. Karreth F. A. Buel G. R. Jedrychowski M. P. Plas D. R. (2017). Focal adhesion- and IGF1R-dependent survival and migratory pathways mediate tumor resistance to mTORC1/2 inhibition. *Mol. Cell.* 67 51–527.e514. 10.1016/j.molcel.2017.06.033 PMC5698809 28757207

[B62] You R. Li X. Liu Y. Liu G. Lu S. Li M. (2014). Response of filopodia and lamellipodia to surface topography on micropatterned silk fibroin films. *J. Biomed. Mater. Res. A* 102 4206–4212. 24464986 10.1002/jbm.a.35097

[B63] Zhang Y. Liu X. Zeng L. Zhang J. Zuo J. Zou J. (2019). Polymer fiber scaffolds for bone and cartilage tissue engineering. *Adv. Funct. Mater.* 29:1903279.

[B64] Zheng G. Guan B. Hu P. Qi X. Wang P. Kong Y. (2018). Topographical cues of direct metal laser sintering titanium surfaces facilitate osteogenic differentiation of bone marrow mesenchymal stem cells through epigenetic regulation. *Cell Prolif.* 51:e12460 10.1111/cpr.12460 PMC6528859 29701270

[B65] Zhu L. Luo D. Liu Y. (2020). Effect of the nano/microscale structure of biomaterial scaffolds on bone regeneration. *Int. J. Oral Sci.* 12:6. 10.1038/s41368-020-0073-y PMC7002518 32024822

