# Supplementary material for: Hierarchical Micro-Nano Topography Promotes Cell Adhesion and Osteogenic Differentiation via Integrin α2-PI3K-AKT Signaling Axis
Source: Front Bioeng Biotechnol. 2020 May 19;8:463. doi: 10.3389/fbioe.2020.00463 (PMC7248375; doi:10.3389/fbioe.2020.00463)
Supplement: Supplementary file 1 [file Table_1.DOCX]

***Supplementary material***


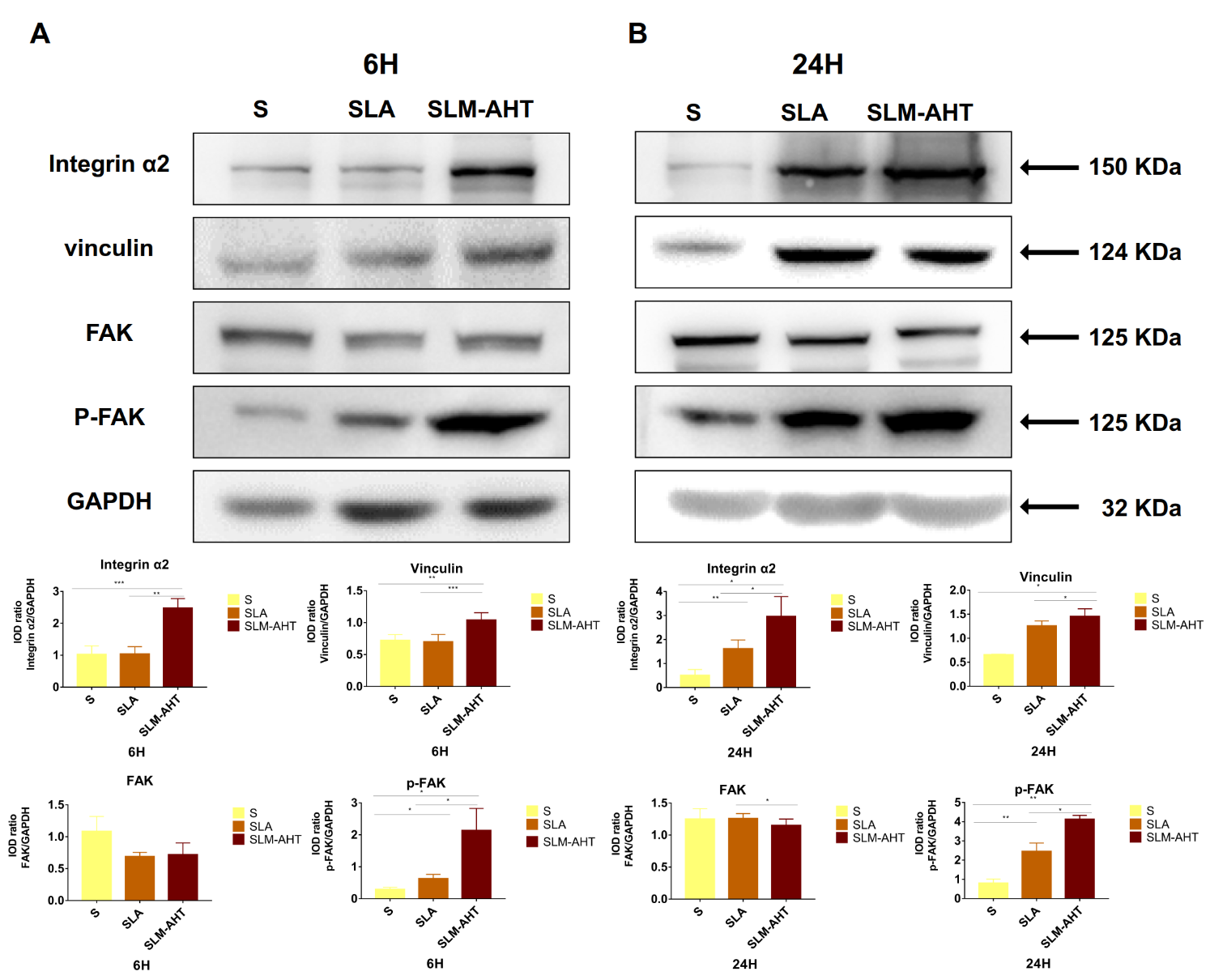


**Supplementary Figure 1: The western blot quantitative analysis results of Figure 4.**

**
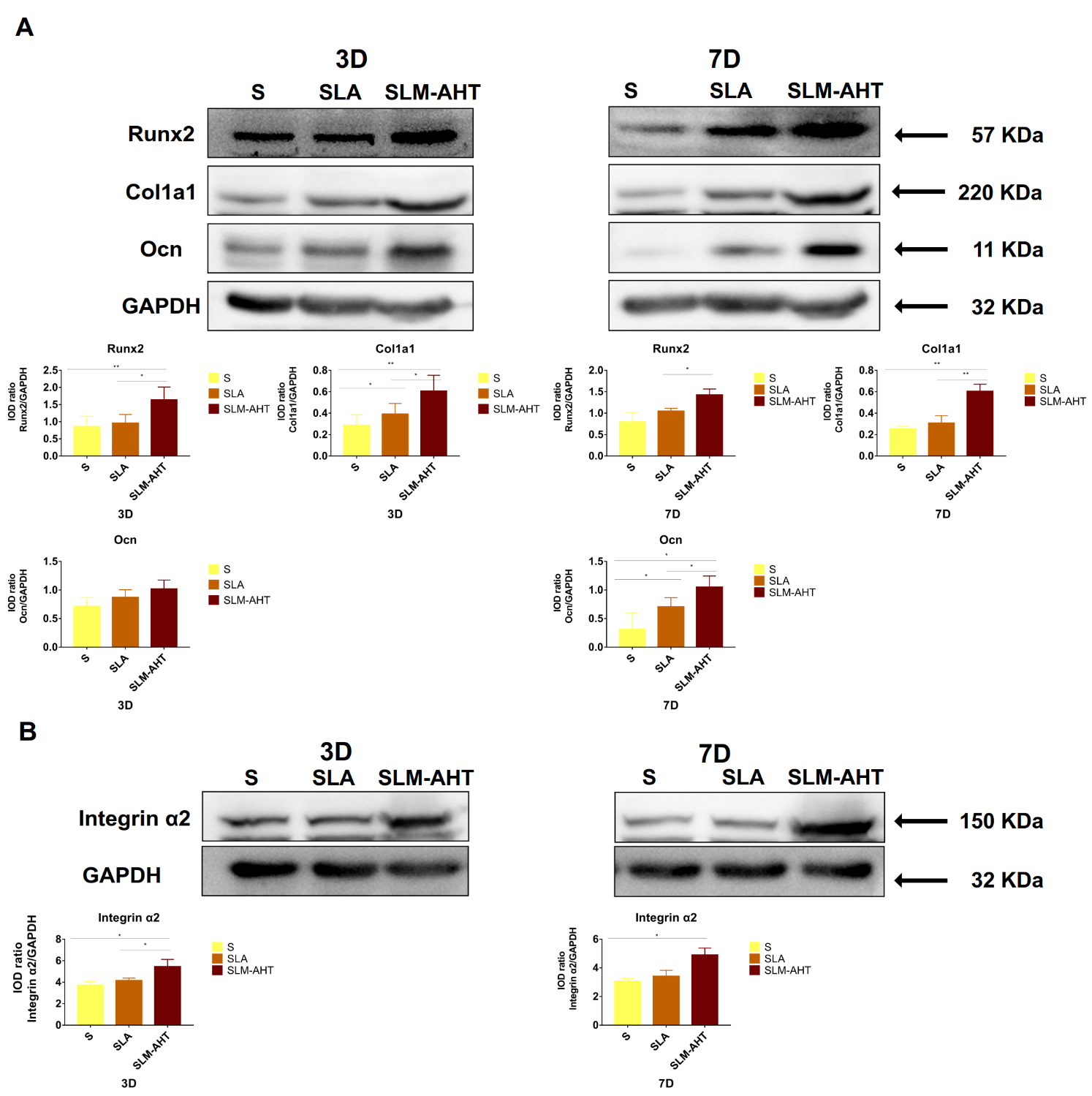
**

**Supplementary Figure 2: The western blot quantitative analysis results of Figure 5.**


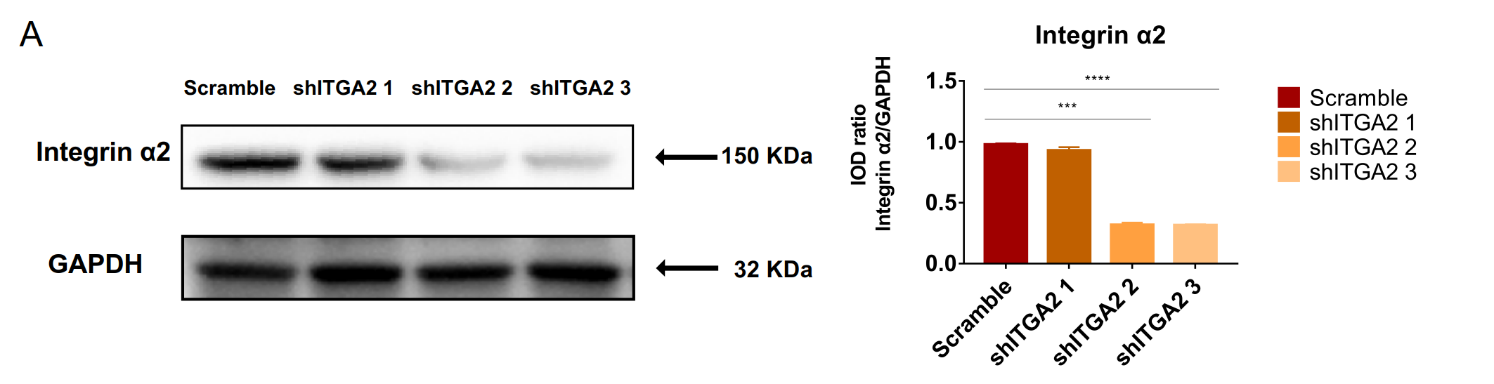


**Supplementary Figure 3: The western blot quantitative analysis results of Figure 6.**

**
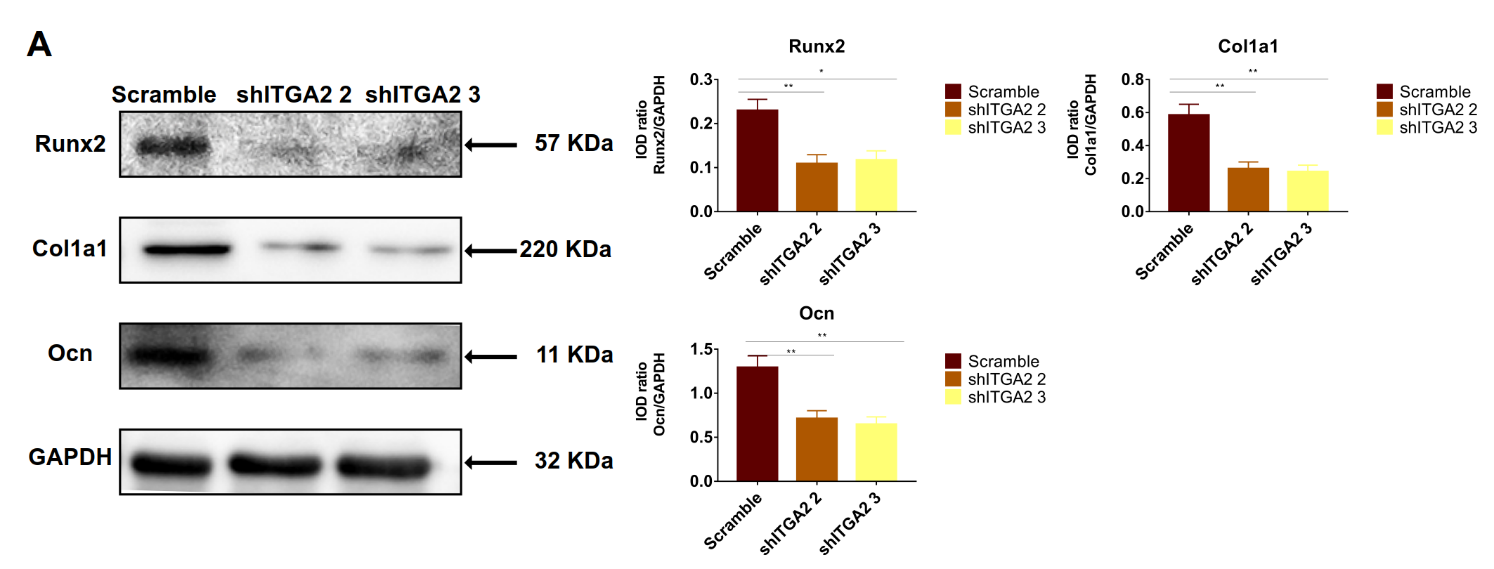
**

**Supplementary Figure 4: The western blot quantitative analysis results of Figure 7.**

**
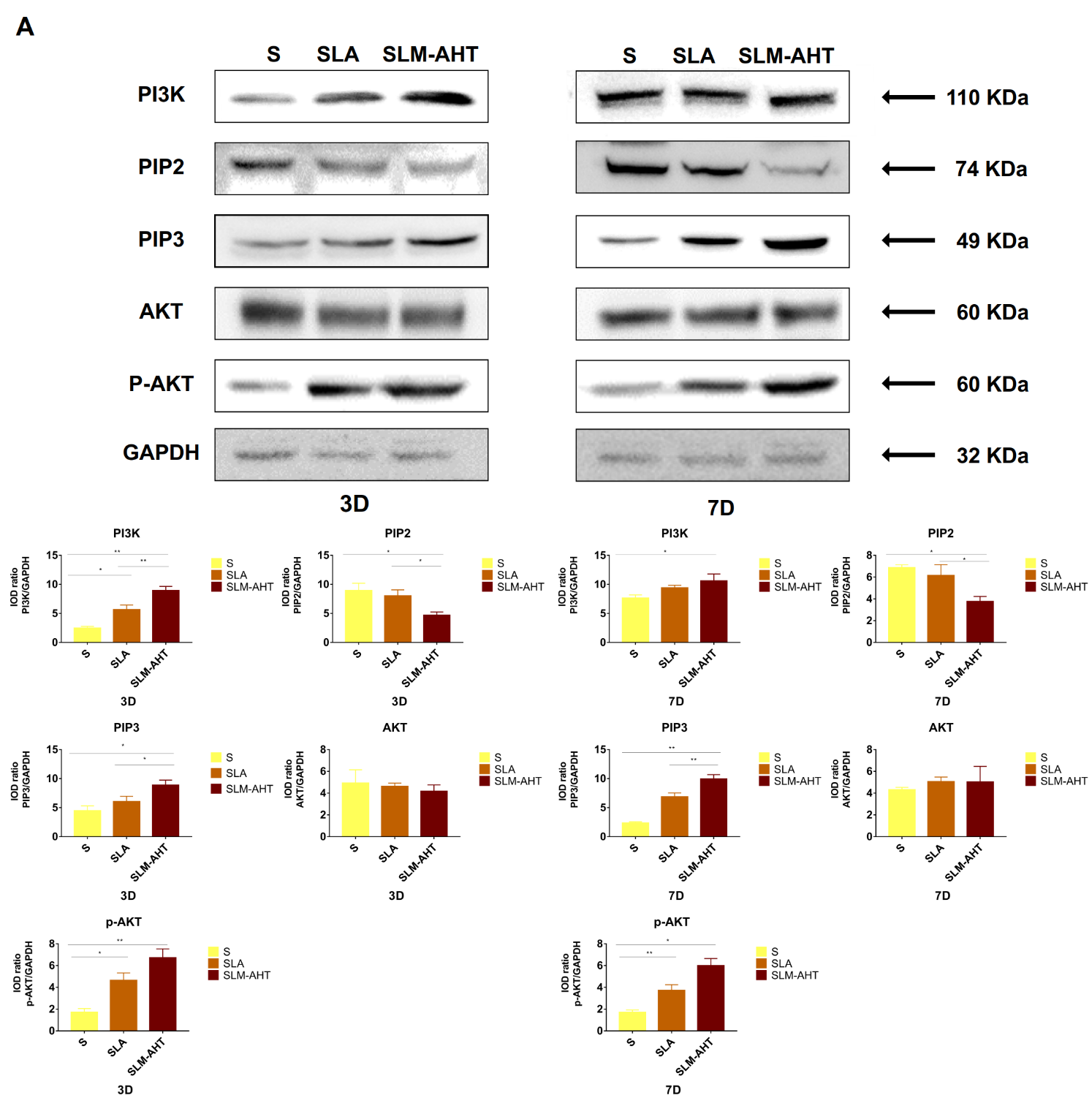
**

**Supplementary Figure 5: The western blot quantitative analysis results of Figure 8.**

**
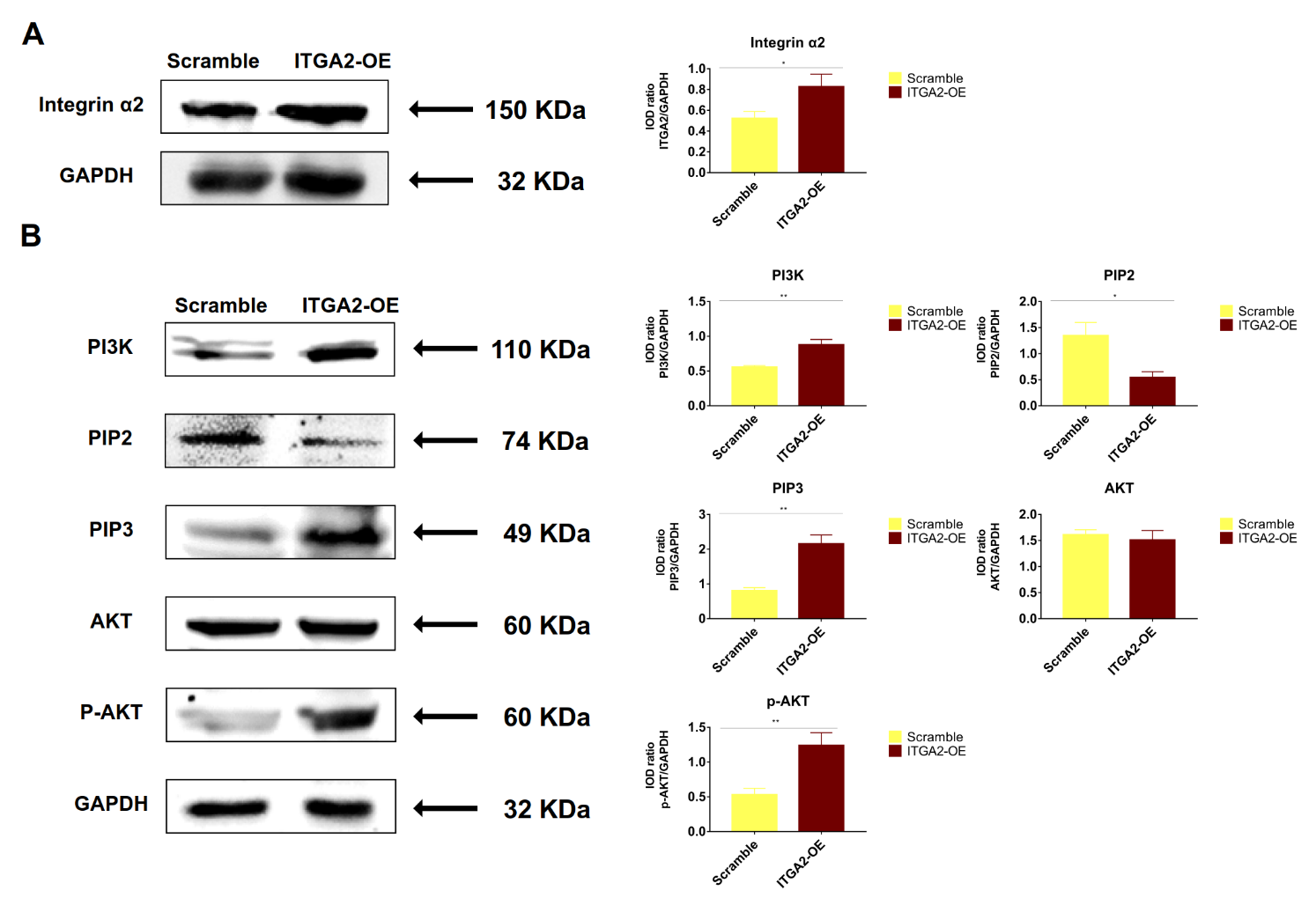
**

**Supplementary Figure 6: The western blot quantitative analysis results of Figure 9.**


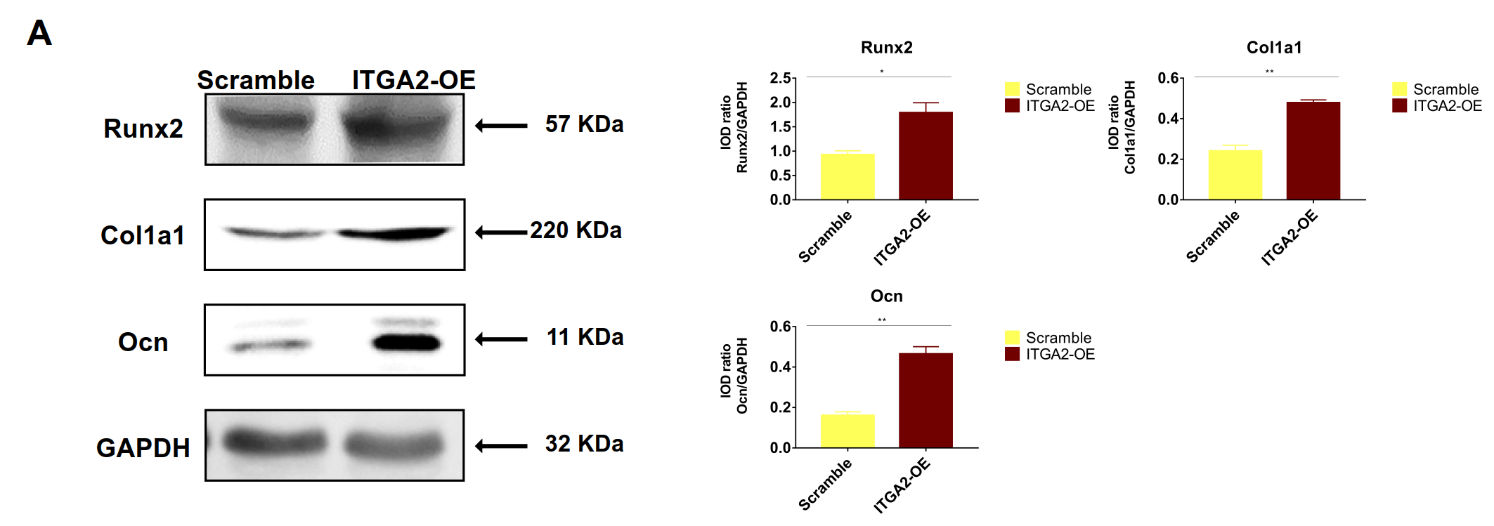


**Supplementary Figure 7: The western blot quantitative analysis results of Figure 10.**
